# Fabrication of carbon black nanoparticles from green algae and sugarcane bagasse

**DOI:** 10.1038/s41598-024-56157-4

**Published:** 2024-03-06

**Authors:** Nehad A. Elmaghraby, Mohamed A. Hassaan, Mohamed A. Zien, Elsayed M. Abedelrhim, Safaa Ragab, Murat Yılmaz, Ahmed El Nemr

**Affiliations:** 1 Environment Division, National Institute of Oceanography and Fisheries (NIOF), Kayet Bey, Elanfoushy, Alexandria, Egypt; 2 Chemistry Department, Faculty of Science, Damanhur University, Damanhur, Egypt; 3 Department of Chemistry and Chemical Processing Technologies, Bahçe Vocational School, Osmaniye Korkut Ata University, Osmaniye, 80000 Turkey

**Keywords:** Chemistry, Chemical engineering, Nanoscale materials

## Abstract

There are several industrial uses for carbon black (CB), an extremely fine powdered form of elemental carbon that is made up of coalesced particle aggregates and almost spherical colloidal particles. Most carbon black is produced from petroleum-derived feedstock, so there is a need to find an alternative method to produce CB, which relies on renewable resources such as algae and agricultural waste. A process involving hydrolysis, carbonization, and pyrolysis of green algae and sugarcane bagasse was developed, as the optimal hydrolysis conditions (16N sulfuric acid, 70 °C, 1 h, 1:30 g/ml GA or SC to sulfuric acid ratio), a hydrolysis ratio of 62% for SC and 85% for GA were achieved. The acidic solution was carbonized using a water bath, and the solid carbon was then further pyrolyzed at 900 °C. The obtained carbon black has a high carbon content of about 90% which is confirmed by EDX, XRD, and XPS analysis. By comparison carbon black from sugar cane bagasse (CBB) and carbon black from green algae *Ulva lactuca* (CBG) with commercial carbon black (CCB) it showed the same morphology which was confirmed by SEM analysis. The BET data, showed the high specific surface area of prepared CB, which was 605 (m^2^/g) for CBB and 424 (m^2^/g) for CBG compared with commercial carbon black (CBB) was 50 (m^2^/g), also the mean pore diameter of CBB, CBG and CCB indicated that CBB and CBG were rich in micropores, but CCB was rich in mesoporous according to IUPAC classification. This study might have created a technique that can be used to make carbon black from different kinds of biomass.

## Introduction

Carbon Black (CB) is a very fine powder of elemental carbon consisting of fused particle aggregates and nearly spherical colloidal particles^1,2^. It typically has an elemental carbon content of over 97%, with varying proportions of sulphur, oxygen, and hydrogen^3^. CB materials are categorized by their impurity profile, their agglomeration and aggregation status, and their primary particle size. It rapidly forms aggregates that are a chain of a few or hundreds of primary carbon particles in the range of 100–800 nm or above shaped as roughly spherical primary particles and fused in a random branching structure. The particle size of CB materials ranges from 10 to 100 nm so it is considered as a nano-size material^4,5^.

Many diverse fields, including electronics and fuel cells^6^, lithium-ion secondary batteries^7^, hydrogen storage^8^, chemical sensors^9^, biosensor^10^, solar generator^1,11^, nanoscale electronic devices and supercapacitors^12^ as a good conductor of electricity use carbon black in a variety of ways. Over 70% of CB application is as a reinforcing phase in automobile tires and pigment. The common use of CB as a pigment in inks, coatings and plastics as it absorbs ultraviolet radiation that causes the material to degrade^13^. It is also used in the coloring of resins and films due to its high tinting strength and stability. On the other side, carbon black particles have applications on radar absorbent materials as they reduce the aircraft radar cross-section and also in photocopiers, laser printer toner, paints, and other inks. The other uses of carbon black are in the production of belts, hoses, and other non-tire rubber goods consume about 20% of the world’s production of CB^14–17^*.* More recently, carbon black has been used in biological applications as antibodies as described in Amornwachirabodee et al.^18^.

About ten million tons of carbon black were produced globally in 2005. The impingement (channel), oil-furnace, acetylene (decomposition), lampblack, and thermal (decomposition of natural gas) processes are the main processes for the production of carbon black. Therefore, carbon blacks have five types; lampblack, thermal black, furnace black, acetylene black, and channel black commonly denoted by the process or the source material from which they are produced^15,19^. The most common one is the furnace type which is made from petroleum-derived feedstock, which produces about 90–95% of carbon black^3,20–24^. Alternative techniques have been researched more recently, although they still rely on nonrenewable raw resources. Examples of these techniques include plasma technology^25,26^, and the use of an aerosol flame reactor^27^. The use of renewable resources, such as rice husk (RH), which is widely available in nations like China, should theoretically make it possible to generate carbon black without requiring petroleum goods^28,29^. In this study, we developed a new method^29^ for the synthesis of carbon black from renewable sources such as green algae and agriculture waste (sugarcane bagasse) and also the resulting carbon black compared with commercial ones.

## Methods

### Materials and reagents

Green algae (GA) (*ulva lactuca*), obtained from Abu Qir, Egypt and Sugarcane bagasse (SC), obtained from marketing, Egypt, were washed thoroughly with pure water to eliminate adhering dust and soil and overnight dried at 70 °C. Analytical grade H_2_SO_4_ (95 wt.%) was obtained from Fisher Scientific, UK, methylene blue (basic blue 9) (C: I-52015) C_16_H_18_N_3_Cl_5_.xH_2_O from honey well Ridel-deHaĕn AG, SEELZE-HANNOVER, Germany, and commercial graphitized carbon black from Bel Japan Inc. certificate for reference materials.

### Preparation of CB

CB was prepared through three steps hydrolysis, carbonization, and pyrolysis process. The first step involved the hydrolysis of SC and GA by sulfuric acid. The hydrolysis conditions were studied at reaction temperatures of 50 to 90 °C, ratios of SC or GA to H_2_SO_4_ of 1:10 to 1:30 (g/ml), and reaction periods of 30 to 180 min. Sulfuric acid concentrations ranged from 1 to 18N. The reactions happened inside a 500 ml conical flask submerged in water. The mixtures were filtered after the reactions were finished and cooled to room temperature. Before being mixed with the filtrate for the second phase, the solid residue was dried at 70 °C overnight and washed with distilled water (DW) to neutrality for weight determination. The second step was the carbonization step by using 500 ml of hydrolysis solution heated at 95 °C for 2 h at the mantel and then filtered through a Buchner funnel. The produced solids were neutralized with DW before being overnight dried at 70 °C. The third step involves pyrolyzing the acquired solid carbon from the second step for an hour at 900 °C under nitrogen gas in a tube furnace. The temperature rose at a 30 °C/min rate.

### Analysis

Fourier transform infrared (FTIR) spectroscopy (using platinum ATR) was utilized to identify the IR-observable functional groups on the carbon surface in the wave number (400–4000 cm^−1^) and estimate the surface functional groups of the CBs^30^. Nitrogen adsorption–desorption isotherms were used to characterize the carbon black’s surface area after it had been degassed in a processor (BELPREP-vac II, made by BEL Japan, Inc.) at 300 °C under vacuum for three hours. Following pre-treatment, the generated carbon black samples’ pore textures were evaluated for adsorption–desorption studies using nitrogen that was 99.99% pure in a porosity analyzer (BELSORP-mini 2, BEL Japan, Inc.). The Brunauer–Emmett–Teller (BET) method was used to determine the specific surface area (*S*_*BET*_), total pore volume (*V*_*T*_), and mean pore diameter (*D*_*P*_) of carbon black. The BELSORP analysis program software was used to calculate the mesopore surface area (*S*_*me*_) and the mesopore volume (*V*_*me*_) of carbon black, as well as the micropore surface area (*S*_*mi*_) and micropore volume (*V*_*mi*_), using the t-plot and Barrett-Joyner-Halenda (BJH) techniques, respectively^31^. Equation (1) was used to get the average pore radius.

1 $$r=\frac{{2V}_{T }({\text{ml}}/{\text{g}})}{{a}_{S,BET}({{\text{m}}}^{2}/{\text{g}})}\times 1000,$$

where, (*r*) is the average pore radius (nm), (*V*_*T*_) is the total pore volume (ml/g), and (*a*_*S,BET*_) is the surface area (m^2^/g). The adsorption–desorption isotherm of nitrogen gas was determined at its boiling point of 77 K^32–34^. The size and the morphology of the samples were examined by using a scanning electron microscope (SEM) (LEO, 1450 vp) under the following conditions: The samples were spread onto carbon stickers on SEM specimen stub. Using a potential difference between 5 and 20 kV, secondary electron imaging micrographs were taken. SEM observations were carried out at magnifications ranging from 160 to 15000x. The elemental composition of carbon black was determined using a (LEO, 1450 vp) Dispersive X-ray (EDX) Analyzer. The morphology and particle sizes of samples were tested by using a Transmission Electron Microscope (TEM) (Jeol-Cx-100). Samples that are made by including test tubes containing 1 cm^3^ of ethanol, and small (8–10 mg) quantities of carbon black were introduced. In a large ultrasound water bath, the caped test tubes were sonicated for three minutes, creating opaque, dark dispersions. A clean TEM specimen grid was given one drop of each, and it was then given a minute to dry. Several photos of each sample were captured after the grid was placed within the transfer chamber of the electron microscope. Depending on the degree of each sample’s dispersion, the magnification ranged from 7500 to 10000x. All of the used magnifications have already been calibrated for the microscope’s magnification factor. On a second generation, Bruker 2D Phaser X-ray diffractometer made by Bruker, Germany, X-ray diffraction (XRD) patterns were acquired at 30 kV using Cu Kα radiation (= 1.540598 Å) and in the range 2θ: 5–80°. Particle size analysis of carbon black samples was done using Malvern IInstruments Ltd, master-sizer 3000 (UK). An ultrasonic magnetic stirrer was used to stir up the samples in water before they were added to the apparatus. The surface chemistry of the CB samples was investigated using XPS analysis using the following method. Monochromatic X-ray Al K-alpha radiation with a spot size of 400 µm, pressure of 10^−9^ mbar, and pass energies of 200 e.V for the whole spectrum and 50 e.V for the narrow spectrum were used to collect XPS data on K-ALPHA (Thermo Fisher Scientific, USA)^35–38^.

### The moisture and ash content analysis

The moisture content of carbon black study was performed by heating 0.5 g of it to 110 °C in a crucible for 24 h. Following this, the weight loss for each sample was noted, and the moisture % was computed using Eq. (2).

2 $$\mathrm{Moisture\, \% }= \frac{W\left(initial\right)-W(final)}{W(initial)} \times 100.$$

The ash content of carbon black were analyzed by heating 0.5 g of samples at 500 °C for 6 h in a crucible. The final weight was then recorded, and Eq. (3) was used to get the ash percentage^39^.

3 $$\mathrm{Ash \,content}=\frac{W({\text{final}})}{W(initial)}.$$

### Bulk density test

Bulk density testing involves taking a certain amount of carbon black into a glass cylinder (10 mL), then filling it to the required level and drying it overnight in an oven set at 80 °C. Following the formula (Eq. (4)), the bulk density was computed and given as g/ml after the cylinder was tapped for 1-2 min to compress the carbon^34^:

4 $$\mathrm{Bulk \,density}=\frac{Weight\, of\, dry \,material(g)}{Volume\,of\, packed \,dry \,material(ml)}x100.$$

### Methylene blue adsorption

The adsorption characteristic of carbon black was investigated by using methylene blue dye as the adsorbate. In order to achieve equilibrium, an aqueous solution containing 0.1 g of the adsorbent and 100 ml of MB dye solutions with original concentrations of 50–200 mg/L were put in glass flasks and shaken for 24 h at 30 °C. An Analytic Jena (SPEKOL300) spectrophotometer operating at 665 nm was used to measure the quantities of MB dye in the supernatant solution both before and after adsorption. Using the Eq. (5), the amount of adsorption at equilibrium, *q*_*e*_ (mg/g), was determined^39,40^.

5 $$q_{{\text{e}}} \, = \,\left( {C_{{\text{i}}} - C_{{\text{e}}} } \right)\, \times \,{\text{V}}/{\text{m,}}$$

where, *C*_i_ is the original concentration, *C*_e_ is the concentration at equilibrium, *V* is the volume of the solution, and *m* is the CB mass.

## Result and discussion

### Characterization of prepared carbon black and the raw materials

#### Electron microscopy

The size and shape of primary particles and aggregates of carbon black in the dry state can be determined directly and accurately using electron microscopy. The standard test method ASTM D 3849-04, which outlines a protocol for preparing microscope specimens, evaluating the samples in transmission electron microscopy, and for interpreting results via image processing software, applies to this procedure. The approach was primarily created to characterize the characteristics of carbon black^41^.

##### SEM of raw materials (GA and SC)

**Figure 1:**
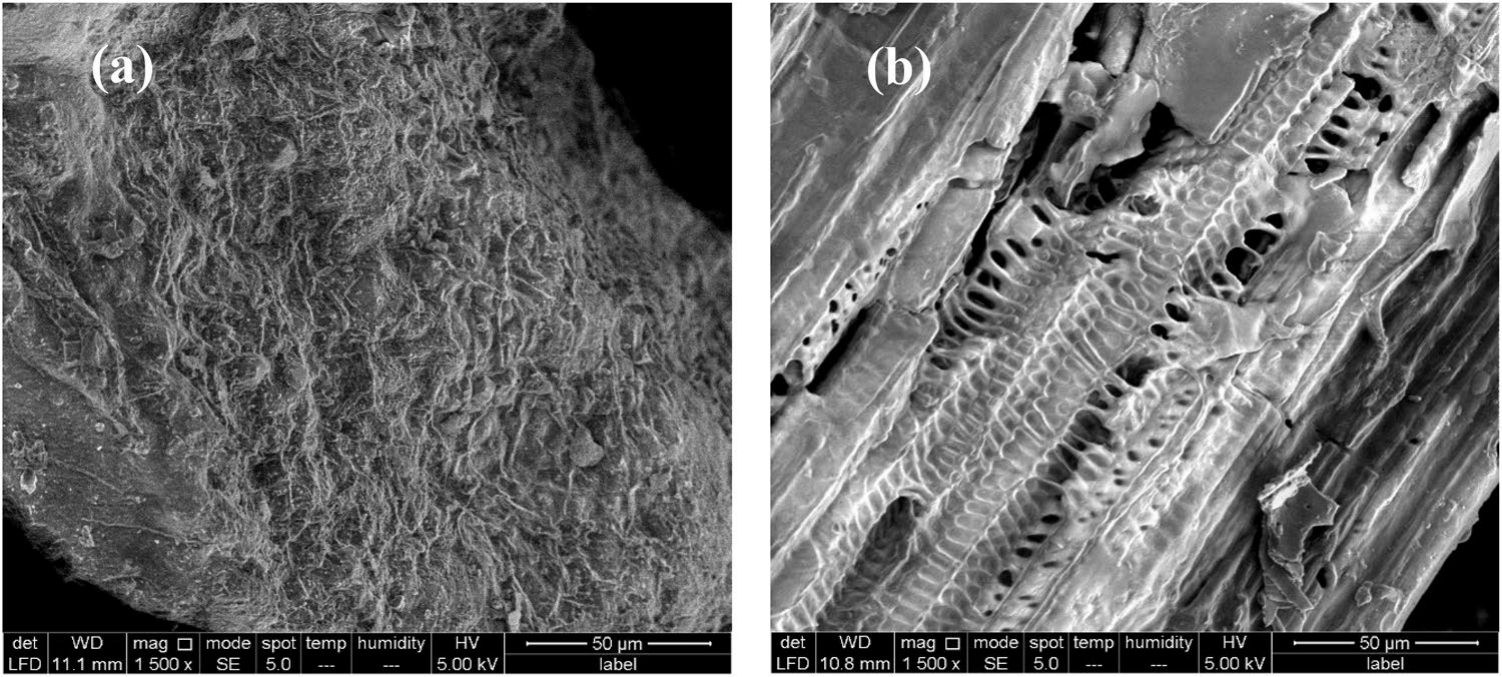
SEM image of raw materials (**a**) sugar cane bagasse (**b**) green alga *Ulva lactuca* using 15 kV and 1500 X magnifications.

The use of scanning electron microscopy proved to be of great versatility and importance for studying biomass structure (Fig. 1a). The sugarcane bagasse sample presents a rigid and compact morphology. The green algae sample shown in Fig. 1b demonstrates the amorphous superficial structure of the algal surface^42–44^.

##### SEM and TEM of CBB and CBG:

**Figure 2:**
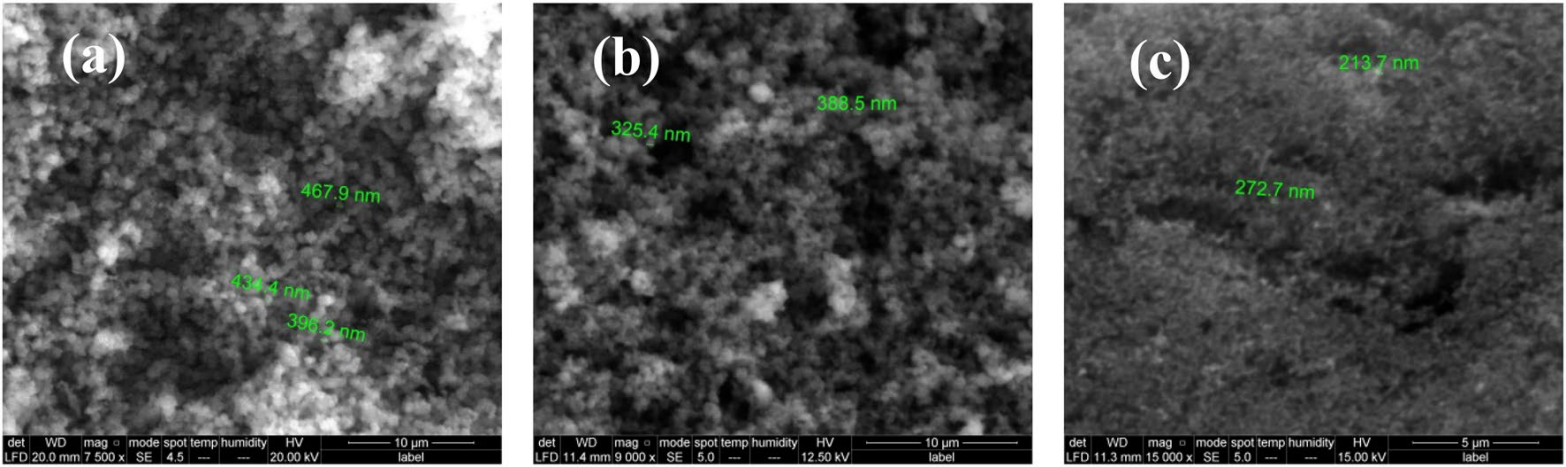
SEM image of carbon black (**a**) carbon black prepared from sugarcane bagasse (CBB), (**b**) carbon black prepared from green algae *Ulva lactuca* (CBG) and (**c**) commercial carbon black using 20, 12.5 and 15 kV and 7500X, 9000X and 15,000 X magnifications.

**Figure 3:**
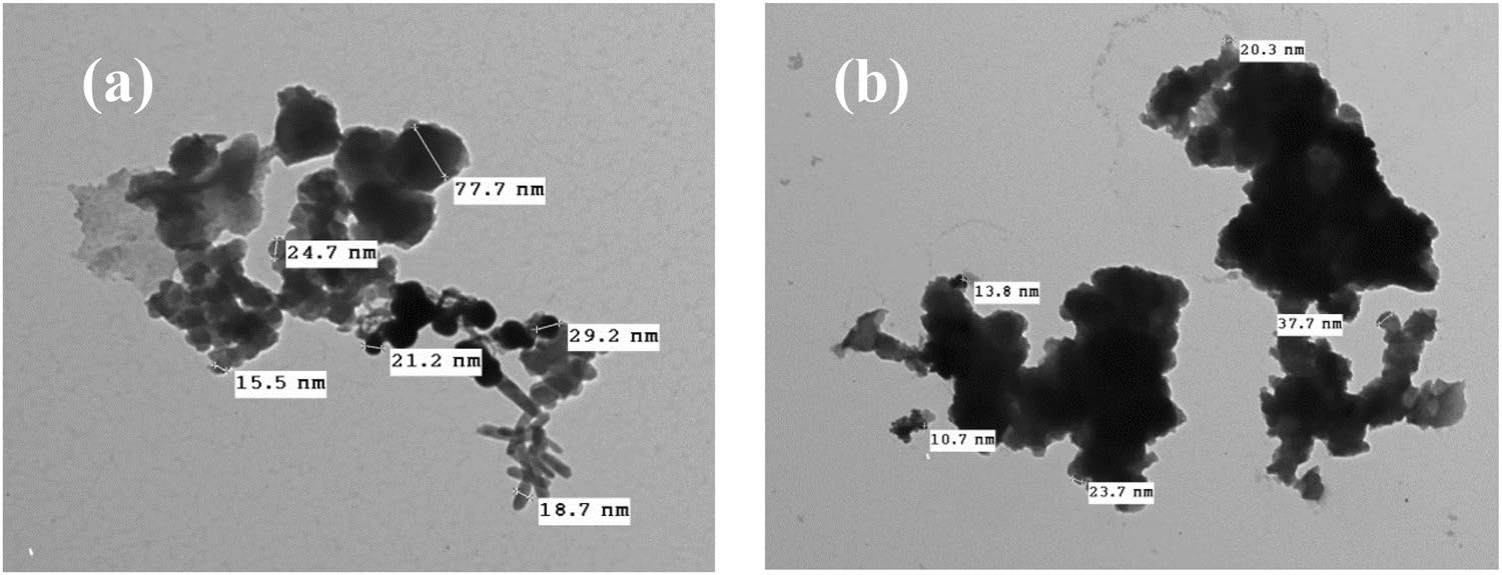
TEM image for carbon black (**a**) carbon black from sugarcane bagasse (CBB) and (**b**) carbon black from green algae *Ulva lactuca* (CBG) using 80 kV and 10000X magnifications.

SEM analysis was used to examine the surface morphology of the carbon black samples. As seen in Fig. 2a,b for CBB and CBG, respectively the carbon black particles form aggregates constituted of spherical-shaped nanoparticles of amorphous carbon. In comparison, the commercial carbon black shows the same morphology made up of aggregates constituted of particles about 140–300 nm in diameter as presented in Fig. 2c. Additionally, the porous surface produced by this morphology can offer a larger electroactive surface area. This observation is in good agreement with the TEM image, as shown in Fig. 3a,b for CBB and CBG, respectively. The TEM images display the prepared carbon black particles in the nanoscale ranged from 18.7 to 77.7 nm for CBB and from 8.65 to 37.7 nm for CBG^45,46^.

#### EDX analysis

##### EDX of raw materials

Table 1 display the chemical compositions of sugarcane bagasse (SC) and the green algae (GA) samples as found by EDX analysis. The results showed that C, Na, Mg, O, and Al were present in each samples (Table 1). More over SC sample has more carbon content 40.19% than GA 29.28%. However, silicon, phosphorus, chloride, potassium, and calcium contents were very low in the SC sample and weren’t found in the GA sample (Table 1). The results of the SC sample obtained in this investigation are comparable to those of other studies on O, H, C, and N elemental analysis, which reported concentrations of 32.05–50.20%, 2.6–7.0%, 43.8–58.30%, and 0.4–6.8%, respectively, in various agricultural wastes.

**Table 1:** The EDX of sugar cane bagasse (SC) and green algae *Ulva lactuca* (GA).

Sample	Element (%)												
	C	N	O	Na	Mg	Al	Si	P	S	Cl	K	Ca	Total
SC	40.19	6.84	37.65	1.08	1.49	0.78	1.34	0.94	2.5	0.7	0.9	5.6	100
GA	29.28	4.22	40.61	0.93	7.47	2.55	–	–	14.94	–	–	–	100

##### EDX of prepared carbon black

The EDX analysis of the prepared CBs obtained from SC and GA at 900 °C pyrolysis temperature and the commercial carbon black (CCB) is summarized in Table 2. The results indicated that the prepared CBB and CBG contains the same elements with relatively the same amounts of the element. A high carbon content above 90% with oxygen are the main constintunets of the prepared CBB and CBG (hydrogen doesn’t appear in EDX due to its low energy).

**Table 2:** The EDX of carbon black from sugar cane bagasse (CBB), carbon black from green algae *Ulva lactuca* (CBG) and commercial carbon black (CCB).

Sample	Element (%)		
	C	O	Total
CBB	91.2	8.8	100
CBG	92.23	7.77	100
CCB	92.99	7.01	100

#### FT-IR spectra

**Figure 4:**
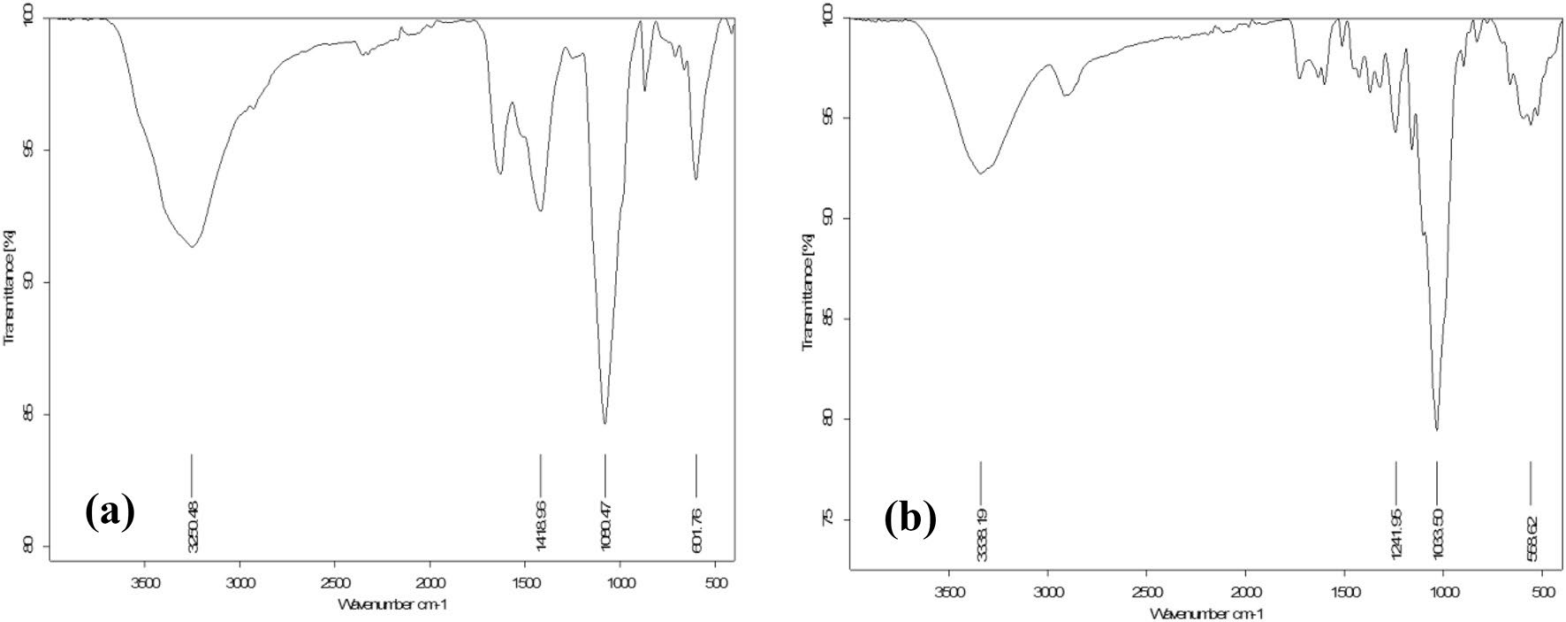
FTIR spectra of raw materials (**a**) green alga *Ulva lactuca* and (**b**) sugarcane bagasse.

In the case of raw material of green algae, Fig. 4a indicates the FT-IR spectra of green algae showed peaks at 3250.48, 1629.48, 1489.96, 1080.47 and 601.76 cm^−1^, the broad intense absorption peak observed at 3350.35 cm^−1^ is due to the presence of stretching vibration of the hydroxyl groups (O–H stretch) on the surface. The peak at 1629.48 cm^−1^ is due to the stretch (C=O) of the carboxylic group. The band of 1418.69 cm^−1^ assigned to (C–H) asymmetric bending vibration of the methyl group ranged from 1500 to 1300 cm^−1^. The sharp peaks around 1080.47 cm^−1^ consider (C–O) stretching, whereas the peaks around 601.76 cm^−1^, represented aromatic (C–H) bending^47^, as well as Fig. 4b shows the FT-IR spectra of sugarcane bagasse displayed peaks at 3338.19, 1600, 1241.95, 1033.50 and 558.62 cm^−1^, the broad intense absorption peak observed at 3338.19 cm^−1^ is due to the presence of stretching vibration of the hydroxyl groups (O–H stretch) on the surface. The peak at 1600 cm^−1^is due to stretch (C=C). The band of 1241.95 cm^−1^ is assigned to (C–O–C) vibration in esters. The sharp peaks around 1033.50 cm^−1^ consider (C–O) stretching, whereas the peaks around 558.62 cm^-1^ represented stretch (C–C) ranging from 400 to 700 cm^−1^^48^.

**Figure 5:**
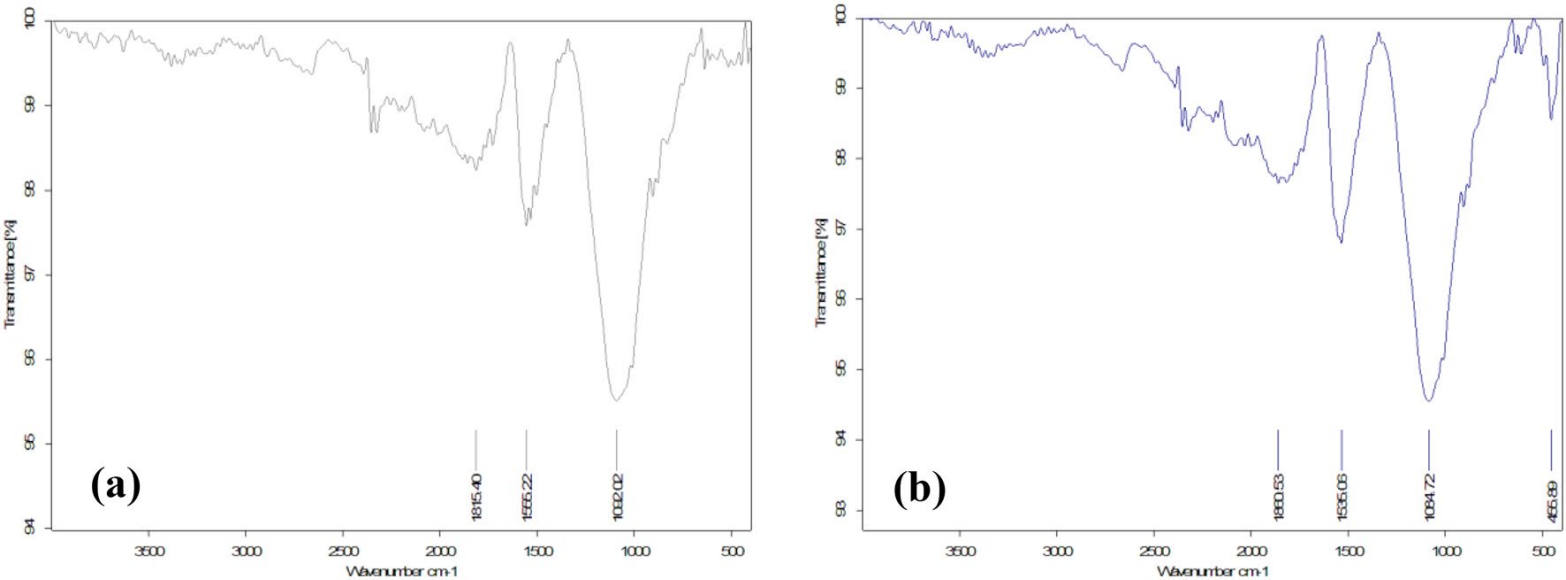
FTIR spectra of carbon black (**a**) carbon black prepared from sugarcane bagasse and (**b**) carbon black prepared from green alga *Ulva lactuca*.

Moreover, Fig. 5 shows the FT-IR spectra of the prepared carbon black from two different raw materials that had the same range of peaks indicating that they have the same functional groups. Peaks at 455.89, and 516.32 cm^−1^ of CBG, and CBB, respectively, were assigned for stretch (C–C). Peaks at 1084.72, and 1092.02 cm^−1^ of CBG, and CBB, respectively, consider for (C-O) stretch bond. Peaks at 1535.06, and 1555.22 cm^−1^ of CBG, and CBB, respectively, were associated with the carbon skeleton (C–C) stretching vibration. The peaks at 1860.53, and 1815.40 cm^−1^ of CBG, CBB, respectively, show two overlap peaks of stretch (C = O) of acid anhydride^49–51^.

#### Nitrogen adsorption–desorption isotherm

**Figure 6:**
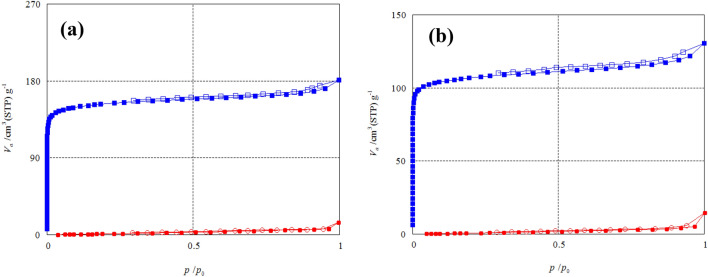
The N_2_ gas adsorption–desorption isotherm plot of (**a**) Raw sugarcane bagasse (SC) and its carbon black (CBB). (filled circle, empty circle: SC, filled square, empty square: CBB) (**b**) Raw green alga *Ulva lactuca* (GA) and its carbon black (CBG). (filled circle, empty circle: GA, filled square, empty square: CBG), where the filled symbols represent the adsorption curve; and hollow symbols represent the desorption curve.

**Figure 7:**
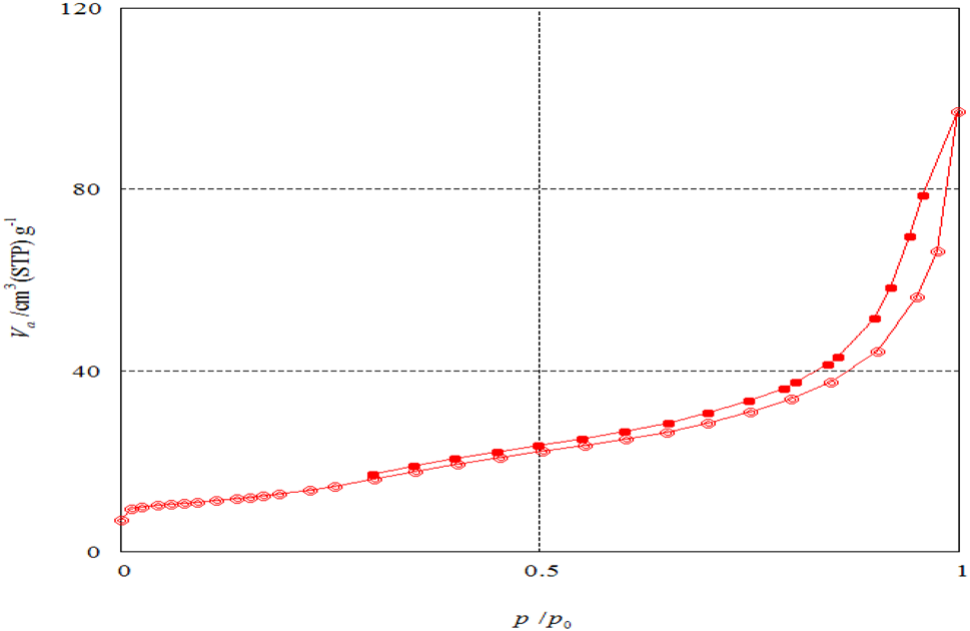
Adsorption–desorption isotherm plot of commercial carbon black (filled circle, empty circle CCB filled symbols represent adsorption curve; hollow symbols represent desorption curve).

**Figure 8:**
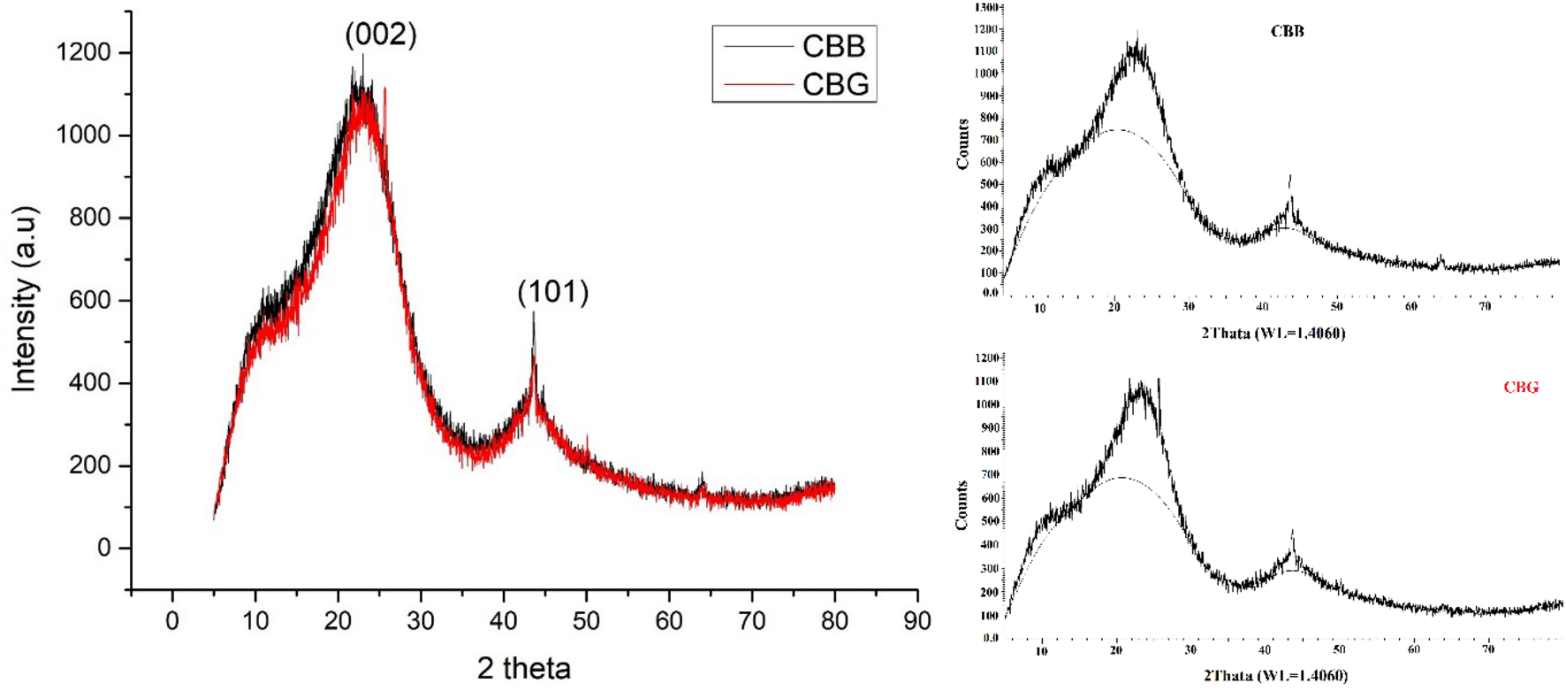
XRD pattern for CBB and CBG.

The N_2_ adsorption–desorption isotherm curves of the two raw materials and their prepared carbon black CBB and CBG are shown in Figs. 6 and 7, respectively. The adsorption–desorption plot of two prepared carbon black CBB and CBG belongs to type (IV) isotherm in accordance with IUPAC isotherm classification, indicating that they were rarely obtained on mesoporous or microporous adsorbents. In contrast, the two raw materials GA and SC’s type (V) isotherm correspond to the IUPAC isotherm classification, indicating that they were rarely obtained on these types of adsorbent. When nitrogen uptake was limited, a steep gradient was seen at the end of the isotherm, indicating capillary condensation in the mesopores. The plateau of this isotherm began at high relative pressures (*P*/*P*_*o*_). The commercial carbon black adsorption–desorption isotherm plot shown in Fig. 8 belongs to type (V) isotherm according to the IUPAC classification indicating that it was uncommonly obtained on microporous or mesoporous adsorbents, provided that the interactions between adsorbent-adsorbate interactions are weak.

The growth of micro- and mesoporous structures on the char during the carbonization process to the nonporous raw materials is indicated by this property, which results in a high adsorption capacity. Capillary condensation in the mesopores of carbon black caused the hysteresis loop^52,53^.

The maximum N_2_ adsorption capacities reach 14.558 and 14.344 cm^3^ g^−1^ for green alga *Ulva lactuca* and sugarcane bagasse, respectively. The maximum N_2_ adsorption capacities reach 124.58, 180.77 and 97.198 cm^3^ g^−1^ for CBG, CBB and CCB, respectively and this indicates that the micropore of CBB > CBG > CCB.

#### Textural properties

The BET surface area (SA) results of the raw GA, SC and their carbon black CBG, CBB were shown in Table 3. The obtained results dispakyed that GA and SC had a very low surface area of 5.2443 m^2^/g and 11.177 m^2^/g, respectively while CBG and CBB had a relatively high surface area of 424.74 m^2^/g and 605.66 m^2^/g, respectively. The results show that the SA of CBB is 605.66 m^2^/g which is higher than the surface area of CBG which is 424.74 m^2^/g, the SA of prepared two carbon blacks (CBB and CBG) is higher than the surface area of commercial carbon black (CBC) that is 50.213 m^2^/g.

**Table 3:** The pore characterization of raw materials (GA and SC), its prepared carbon blacks (CBB and CBG) and commercial carbon black (CBC).

Sample	*a*_*s, BET*_ (m^2^ ∕g)	Mean pore diameter (nm)	Total pore volume (*P*/*Po* = 0.990)	*V*_*mi*_ (cm^3^∕g)	*V*_*me*_ (cm^3^∕g)	*a*_*mi*_ (m^2^/g)
CBB	605.660	1.8285	0.2769	0.2498	0.0271	672.02
CBG	424.740	1.8752	0.1991	0.1726	0.0265	467.16
CCB	50.213	10.6690	0.1339	0.0054	0.1285	4.21
GA	5.244	13.6380	0.0788	3.92E-05	0.0788	–
SC	11.177	7.0788	0.1978	0.0019	0.1959	–

*a*_*s, BET*_ total surface area, *V*_*mi*_ micropore diameter, *V*_*me*_ mesopore diameter and *a*_*mi*_ micropore surface area.

The total pore volumes and average pore diameters of the carbon black prepared from the SC and GA are displayed in Table 3. The mean pore diameter of the two prepared carbon black, CBB and CBG, were 1.828 and 1.8752 nm, respectively which is less than 2 nm according to the IUPAC classification of mean pore diameter, they were rich in micropores and the CCB was rich in mesoporous the mean pore diameter was 10.669 nm. Micropores contributed about 93.319% achieved for the CBB sample and about 92.645% were achieved for the CBG sample. However, the mean pore diameters of the two raw materials were 13.638 and 7.0788 nm of GA and SC, respectively, which means that they were rich in mesopores.

#### XRD patterns

XRD patterns data of the two prepared carbon black are shown in Fig. 8. The prepared carbon black exhibits broad diffused peaks at 24.12, 25.643 and 43.618, 43.612 degrees 2θ for CBB, and CBG, respectively. The dominant intense peak (002) around 25°arises from X-rays diffracted by graphitic planes in the CB. As well as the second peak (100)/(101) around 43° 2θ corresponds to the reflections of the graphitic fraction of CB. The crystalline percentage of the prepared carbon black reached 19.3% and 21.3% for CBB and CBG, respectively, indicating the amorphous carbon structure for carbon black^54,55^.

#### X-ray photoelectron spectroscopy (XPS)

**Figure 9:**
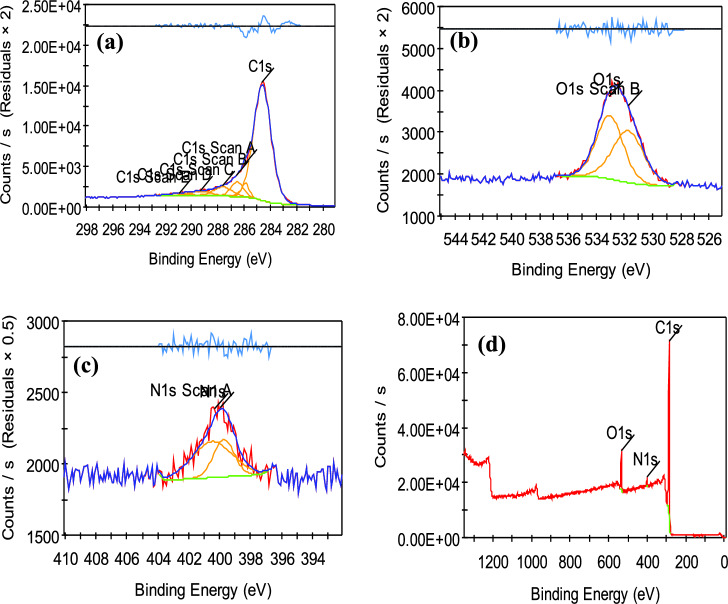
XPS spectra for (**a**) C1s, (**b**) O1s (**c**) N1s and (**d**) survey of carbon black prepared from green algae (CBG).

**Figure 10:**
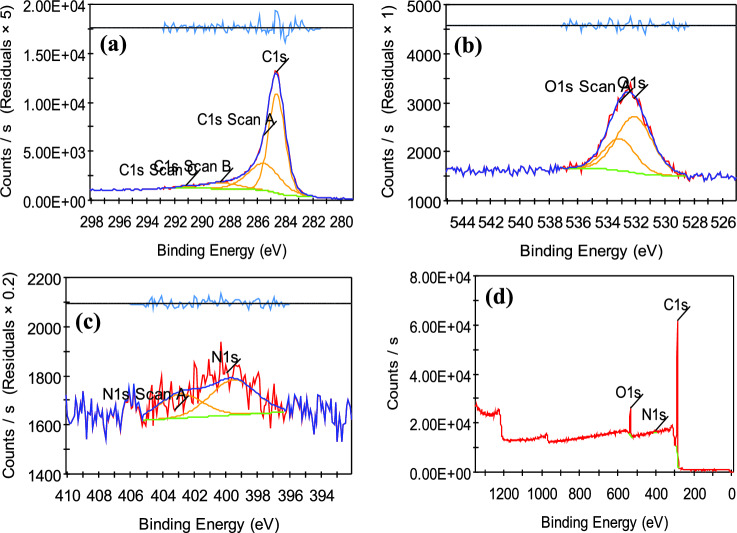
XPS spectra for (**a**) C1s, (**b**) O1s (**c**) N1s and (**d**) survey of carbon black prepared from green algae (CBG).

XPS analysis was achieved on the two prepared carbon black to obtain information about their chemical composition to ensure the purity of the prepared carbon black. The main peak (~ 285.5 eV) is ascribed to the C1s, and the other peaks are ascribed to O1s (~ 533.2 eV), and N 1s (~ 401 eV), respectively. The XPS spectra shown in Figs. 9, 10 indicate that the same elements were found in the two prepared carbon black with approximately the same percentage. The result from Table 4 showed that the two samples contain about 90% carbon and 8% oxygen and also traces of nitrogen reached 2%. These results agree with the result obtained from the previous analysis of EDX that confirmed the successful method for the synthesis of carbon black from sugarcane bagasse and green algae with high purity and high carbon content^56^.

**Table 4:** Carbon XPS spectra.

Sample name	CBB		CBG	
Element	BE (ev)	At (%)	BE (ev)	At (%)
N	285.54	89.89	285.55	89.79
C	533.28	8.30	533.26	8.19
O	401.16	1.81	401.00	2.02

Curve fitting of the C1s photopeaks of the XPS spectra of the prepared CBs is described in Fig. 9a, Table 5 which shows the peak at 284.6, (285.92–285.55), 286.53, (287.6–288.66), (290.89–291.16), 289.26 e.v BE of C-C, C-H, C = O, C = C and COOH, respectively. The curve fitting of the O1s photopeak of the produced carbon blacks’ XPS spectra is similar. Figure 9b shows the presence of two different oxygen surface groups: C = O (BE: 531.7- 532.08 eV), and C-O (BE: 533.03–533.11 eV)^57^. The function group found in the XPS analysis was the same as obtained in the FT-IR analysis. The primary sp3 C signal in the two prepared samples indicates amorphous carbon, while two faint oxygen signals indicate oxide contamination. The two prepared samples show similar fitting peaks. As a result of oxygen being bound to incomplete carbon bonds on carbon black during the synthetic process, the most dominating oxygen peak in CBB is the C–O peak. Since C–O functional groups predominate in all CBs, the quantity of C–O groups in carbon black grows as their specific SA increases. On the side, increasing COOH and COO group content was found to not affect the specific SA in the CB. As a result, this explains why the specific area of CBB is greater than CBG^58^ (Fig. 10).

**Table 5:** Chemical groups on the prepared CBs obtained from curve fitting of the C1s photopeak.

Sample	Functional group of the CBs surface											
	C–C		C–H		C–O		C=O		C=C		COOH	
	BE	At%	BE	At%	BE	At%	BE	At%	BE	At%	BE	At%
CBB	284.6	78.87	285.92	2.74	286.53	7.04	287.60	6.53	290.89	1.48	289.26	3.34
CBG	284.6	59.58	285.55	31.60	–	–	288.66	8.03	291.16	0.79	–	–

#### Particle size analysis

**Figure 11:**
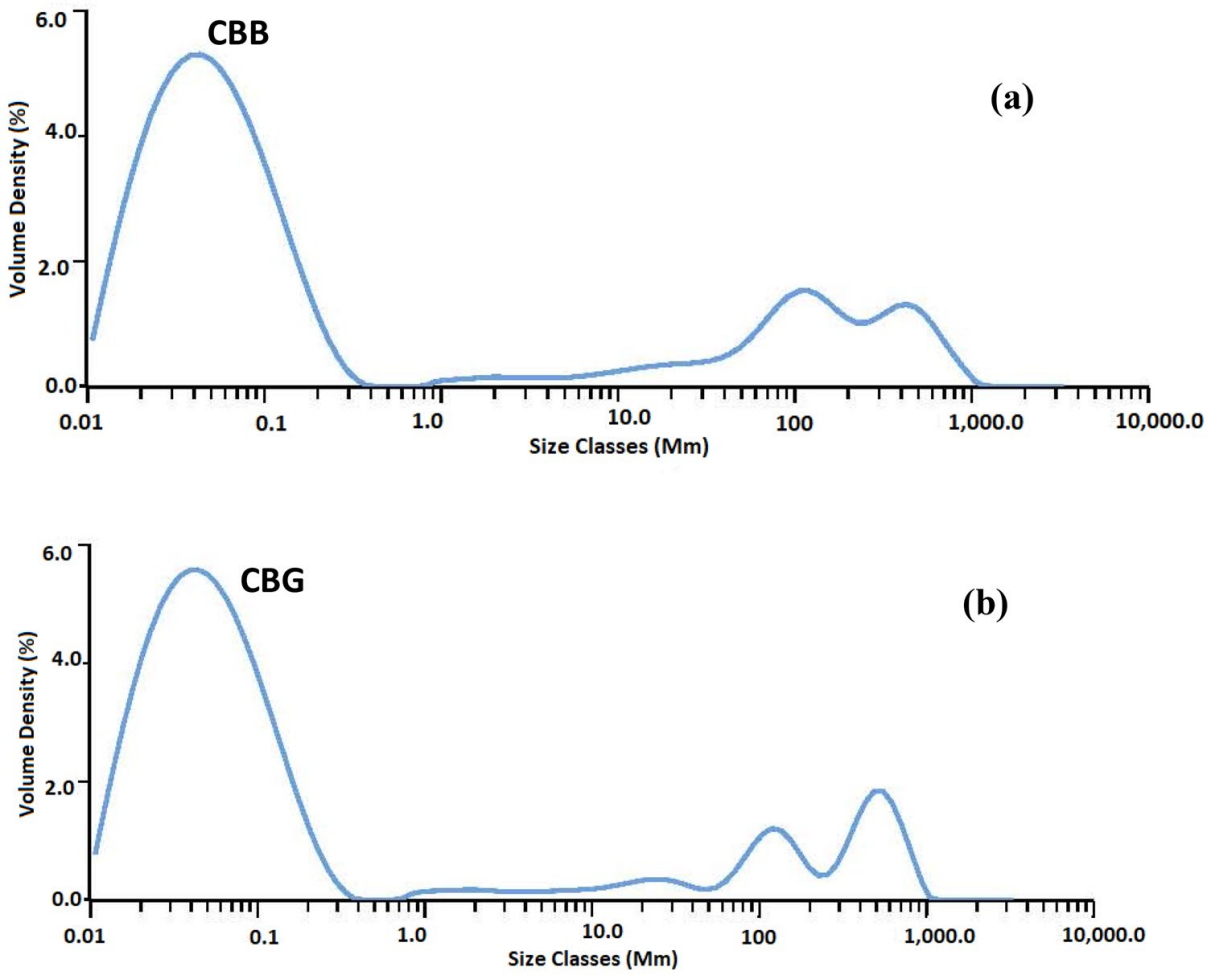
Particle size distribution of CBB (**a**) and CBG (**b**) measured using a Malvern analyzer.

CBG and CBB samples were dispersed in water using an ultrasonic magnetic stirrer before being fed into the equipment, which performed a particle size measurement on the powder samples of carbon black. Utilizing laser diffraction is based on the idea that objects traveling under a laser beam would scatter light at an angle that is precisely proportional to their size. The rate of diffusion and adsorption increases with decreasing porous carbon particle size. Because of the shorter mass transfer zone as the particle size is decreased, there is less intra-particle diffusion, which accelerates the rate of adsorption. Since the carbon black was prepared in a powdered form, it has great efficiency for absorption^42^. Figure 11 shows that in sample CBB, there are about ten percent of the particle size of CBB is less than 0.0199 μm about fifty percent of the particles size of CBB is less than 0.0687 μm and in sample CBG, there are about ten percent of the particles size of CBB are less than 0.0195 μm and about fifty percent of particles size of CBB are less than 0.0636 μm. For Dv_(90)_ the particle size is large due to the accumulation of carbon black, so it can be negligible.

### Physicochemical characterization

#### Ash content, moisture content and bulk density for raw material and the prepared carbon black

When the carbonaceous components are burned up, the surface of carbon is left behind. Ash is a non-carbon or mineral addition that does not chemically interact with this carbon surface. It consists of various undesired mineral substances, which comprise 1–20% and primarily depend on the type of raw material. Since it decreases the mechanical strength of carbon, boosts hydrophilicity, and has an impact on adsorptive capacity, high ash concentration is not ideal for carbon black. The moisture content of carbon, which ranges from 1 to 10%, does not affect its ability to absorb substances but dilutes them^32^.

The prepared carbon blacks had a low content of ash < 10% and also a low moisture content of < 5% due to its high content of carbon. The prepared CBG has a higher ash and moisture content than CBB. This may be due to the ash and moisture content of raw green algae being higher than sugarcane bagasse as shown in Table 6. In addition, it has been determined that the carbon blacks obtained have less ash than the raw green alga and sugarcane bagasse used^59^.

**Table 6:** The physicochemical characterization of the prepared carbon black and raw materials.

Sample	Physicochemical parameter		
	Ash%	Moisture %	Bulk density
GA	35.12	15.30	0.82
SC	2.34	6.56	0.18
CBB	1.32	2.76	0.32
CBG	9.28	4.80	0.30

Floatability of the adsorbent can be seen from above thanks to the carbon’s bulk density, which is a crucial carbon attribute. It implies that adding carbon black to water will cause it to sink, improving contact with the adsorbate and facilitating an efficient adsorption process. In this study, CBB showed a higher bulk density (0.32 g/ml) than^34^ CBG (0.30 g/ml), while, the bulk density of raw sugarcane bagasse is less than raw green alga in Table 6.

### Methylene blue test

The methylene blue number refers to carbon black’s ability to absorb molecules of similar size to methylene blue. The percentage removal of methylene blue after 24 h for initial concentration of 50 and 100 mg/L indicate that the adsorption capacity of CBG is higher than CBB for removal of methylene blue (Table 7). While initial concentration of methylene blue 150 and 200 mg/L showed the same adsorption capacity for CBB and CBG, where the maximum amount of methylene blue adsorbed from each solution were the same 4.24 mg/g for both prepared carbon black^40,60,61^.

**Table 7:** Removal percentage of different concentrations of methylene blue by different CB (CBB and CBG) after 24h.

*C*_0_ (mg/L)	Sample			
	CBB		CBG	
	*C*_*e*_ (mg/L)	*q*_*e*_ (mg/g)	*C*_*e*_ (mg/L)	*q*_*e*_ (mg/g)
50	45.49	4.51	42.75	7.25
100	89.86	10.14	86.06	13.94
150	132.82	17.18	132.82	17.18
200	151.76	48.24	151.76	48.24

### Factors affecting hydrolysis of green alga *Ulva lactuca* (GA) or sugar cane bagasse (SC)

The ratio of GA or SC mass (g) to sulfuric acid volume (mL), reaction temperature, and reaction duration are only a few of the variables that can affect how much GA or SC is being dissolved in sulfuric acid and how much of the reaction is taking place. The hydrolysis percent is defined as the percent of *M*_*l*_/*M*_*G*_ or *M*_*1*_/*M*_*S*_, in which *M*_*l*_ represents the weight loss of GA or SC, *M*_*G*_ represents the initial mass of GA, and *M*_*S*_ represents the initial mass of SC. Since lignin, lipid, protein, hemicellulose, and minerals made up practically the whole solid residue of GA or SC, the statistics on the hydrolysis ratio also included the ash content.

#### ***Impact of H***_***2***_***SO***_***4***_*** concentration on the hydrolysis of GA and SC***

**Figure 12:**
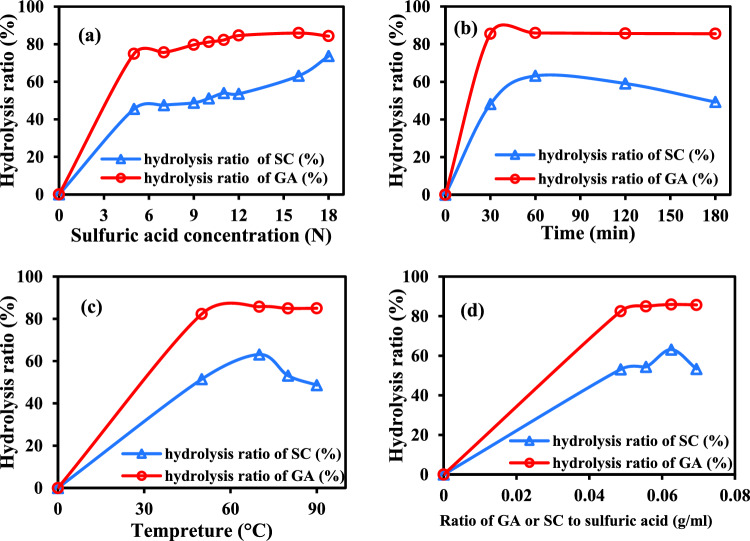
Effect of sulfuric acid (**a**) time (**b**) temperature (**c**) ratio of GA to H_2_SO_4_ (**d**) on hydrolysis percent of green alga *Ulva lactuca* and sugar cane bagasse.

When the other three parameters (hydrolysis temperature, time, and the ratio of GA or SC to sulfuric acid) were fixed at 70 °C, 60 min, and 1:30 (w/v), the hydrolysis % affected by various concentrations of H_2_SO_4_ (5–18N) is presented in Fig. 12a. The hydrolysis percent of GA and SC increased as the acid concentration increased till 16N further increased to 18 N the carbonization occurred, so 16 N was chosen as the optimum concentration of sulfuric acid. The maximum hydrolysis ratio reached 85.91 and 63% for GA and SC, respectively.

#### Impact of time on hydrolysis of GA or SC

According to Fig. 12b, varying hydrolysis periods (60–180 min) had an impact on the hydrolysis percentage. While the remaining parameters (temperature, sulfuric acid concentration, and the GA/sulfuric acid ratio) were fixed at 70 °C, 16 N, and 1:30 (w/v), up to 60 min, the hydrolysis percentage fell. Therefore, 60 min was the best time for further experiments with the maximum value of the hydrolysis percent 85.9% for GA and 63% for SC.

#### Impact of temperature on hydrolysis on GA

The impact of temperature on the hydrolysis ratio of GA or SC is depicted in Fig. 12c. When the other three factors-hydrolysis time, H_2_SO_4_ concentration, and the ratio of GA or SC to sulfuric acid-were fixed at 60 min, 16 N, and 1:30, respectively, the hydrolysis percent increased with rising temperature up to 70 °C. At 70 °C, a maximum hydrolysis ratio of 85.8% for GA and 62% for SC was reached; lower temperatures resulted in incomplete hydrolysis. Carbonization took place above this temperature.

#### ***Impact of ratio of GA to H***_***2***_***SO***_***4***_*** (w/v) on the hydrolysis of GA***

Figure 12d shows the hydrolysis % that is impacted by various GA or SC to sulfuric acid ratios (g/mL). When the other three variables- H_2_SO_4_ concentration, temperature, and hydrolysis time held constant at 70 °C, 16 N, and 60 min, the hydrolysis % achieved its maximum value. Higher GA or SC/H_2_SO_4_ ratios led to carbonization, lower ratios, and an insufficient amount of hydrolysis. Thus the optimum conditions of hydrolysis were 70 °C, 16 N, 60 min and 1:30 (w/v) for temperature, H_2_SO_4_ concentration, hydrolysis time and the ratio of GA or SC to sulfuric acid, respectively. Under these conditions, the GA hydrolysis ratio reached 85.9% and the SC hydrolysis ratio reached 63%.

### % yield of each three production process

The hydrolysis process recognized for the hydrolysis of the cellulosic materials into their monomers (glucose) depends on the amount of cellulosic content in the raw materials, the raw sugarcane bagasse has more cellulosic materials than green algae, so the hydrolysis yield of SC is more than GA. Under ideal hydrolysis conditions, 60 g of sugarcane bagasse yields 20 g of hydrolysis solution, whereas 60 g of raw green alga yields 15 g of hydrolysis solution. The pyrolysis process including transformation from glucose into carbon involved the releasing of O and H atoms as CO, H_2_O, CH_4_, CO_2_, aldehydes or distillation of tar.

The dehydration (carbonization) process yield of hydrolysis solution from green alga is higher than that from sugarcane bagasse. 20 g of hydrolysis solution from sugarcane bagasse gives 8 g carbon, while 15 g of hydrolysis solution from green alga gives 9.35 of carbon. The pyrolysis process increases carbon content and depends on the amount of carbon removed by binding with O and H atoms. The yield of this process was the same for the carbon of the two raw materials shown in Table 8.

**Table 8:** Yield percent of hydrolysis, dehydration (carbonization) and pyrolysis steps of production of carbon black.

Preparation Step	Yield (%)	
	CBB	CBG
Hydrolysis	33.30	25.00
Dehydration (carbonization)	40.00	62.33
Pyrolysis	50.00	50.00

## Conclusion

This study showed the successful method for the synthesis of carbon black from sugarcane bagasse and green algae by hydrolysis, pyrolysis, and carbonization process. The prepared carbon black compared with the commercial carbon black that was the same morphology. The surface area of CBB is 605.66 m^2^/g which is higher than the surface area of CBG which is 424.74 m^2^/g, and these two samples surface area is higher than CBC that is 50.213 m^2^/g. Thus the optimum conditions of hydrolysis were 70 °C, 16 N, 60 min and 1:30 (w/v) for temperature, H_2_SO_4_ concentration, hydrolysis time and the ratio of GA or SC to sulfuric acid, respectively. The composition of carbon black was confirmed by IR, EDX, XRD, and XPS indicating the purity of the prepared carbon black with high carbon content. Moreover, the TEM and particle size analysis of the prepared carbon black indicated that they were in the nanoscale.

## Data Availability

Upon request to the corresponding author of the study, the datasets utilized in this inquiry are available for inspection.
